# Supplementary figures and images for: Quantitative proteomic analysis of skeletal muscles from wild-type and transgenic mice carrying recessive Ryr1 mutations linked to congenital myopathies
Source: eLife. 2023 Mar 2;12:e83618. doi: 10.7554/eLife.83618 (PMC10038659; doi:10.7554/eLife.83618)

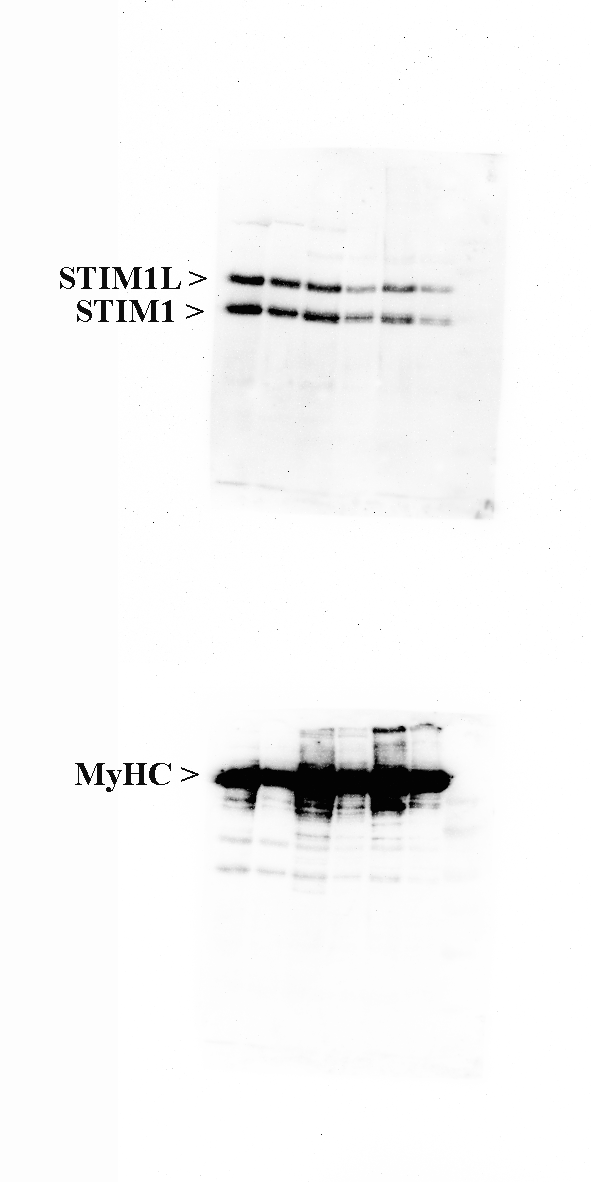

Supplement: Figure 6—source data 1. — TOP: Original uncropped western blots showing immunoreactivity of STIM1L and STIM1 in EDL, Soleus and EOM muscles from WT mouse N° 5. BOTTOM: same blot re-probed with anti-MyHC recognizing all isoforms (loading normalization). [file elife-83618-fig6-data1.zip › Figure_6-source_data_1/ Figure 6_source data 1.tif]

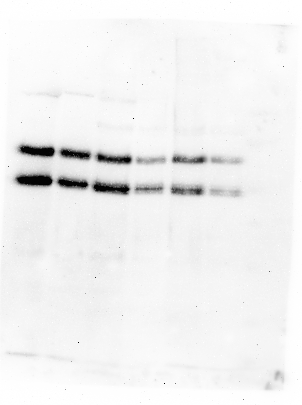

Supplement: Figure 6—source data 1. — TOP: Original uncropped western blots showing immunoreactivity of STIM1L and STIM1 in EDL, Soleus and EOM muscles from WT mouse N° 5. BOTTOM: same blot re-probed with anti-MyHC recognizing all isoforms (loading normalization). [file elife-83618-fig6-data1.zip › Figure_6-source_data_1/stim1 in muscles.tif]

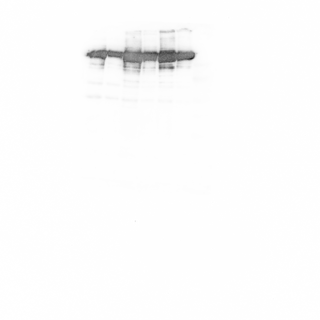

Supplement: Figure 6—source data 1. — TOP: Original uncropped western blots showing immunoreactivity of STIM1L and STIM1 in EDL, Soleus and EOM muscles from WT mouse N° 5. BOTTOM: same blot re-probed with anti-MyHC recognizing all isoforms (loading normalization). [file elife-83618-fig6-data1.zip › Figure_6-source_data_1/ChemiM 1m 20220106 1335.tif]

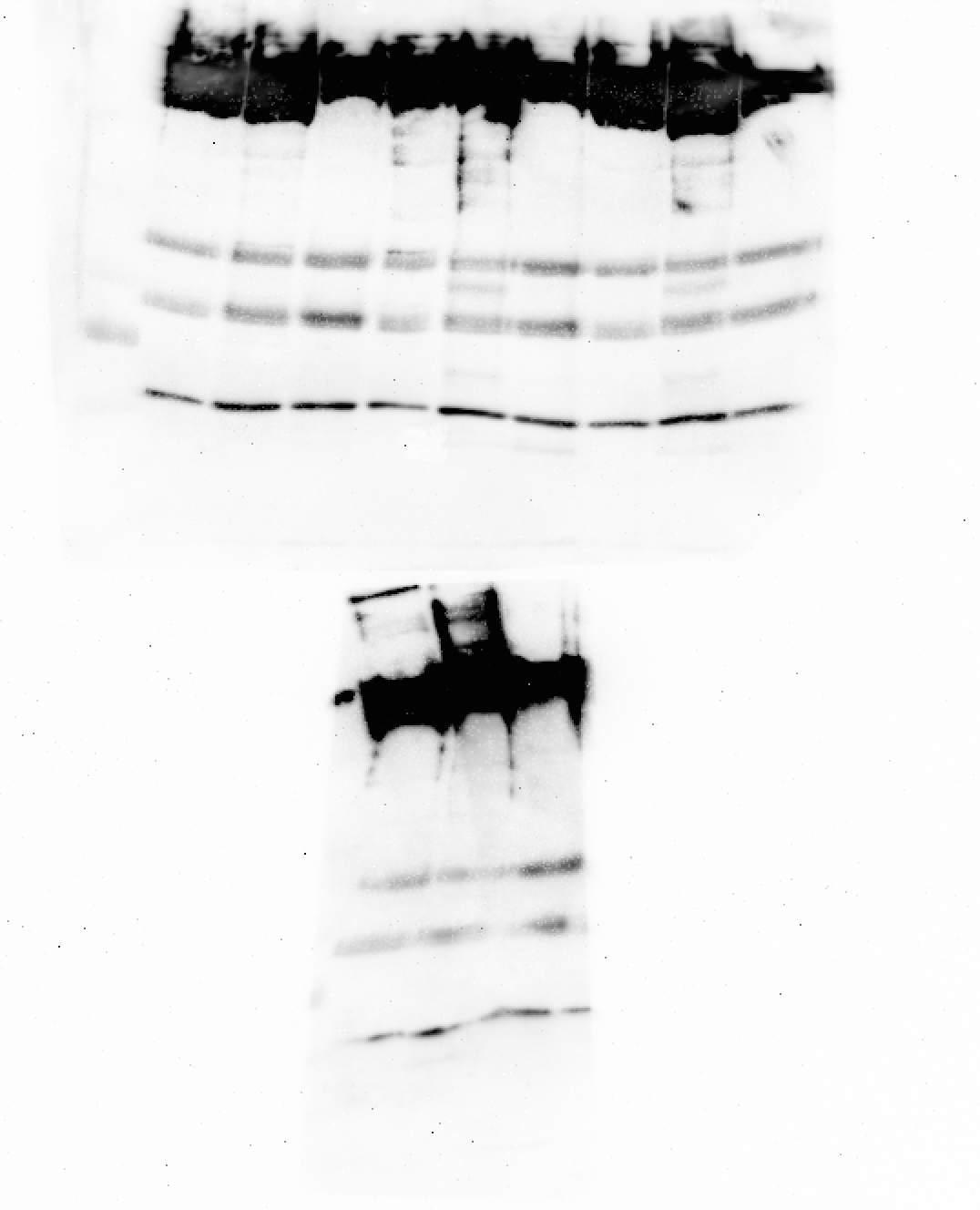

Supplement: Figure 6—source data 2. — Original uncropped western blots showing immunoreactivity of STIM1L, STIM1 and MyHC in EDL, Soleus and EOM muscles from WT mouse N° 1, 2, 3 (TOP) and WT mouse N° 4 (BOTTOM). [file elife-83618-fig6-data2.zip › Figure_6-source_data_2/ChemiM 15m 20220111 1528.png]

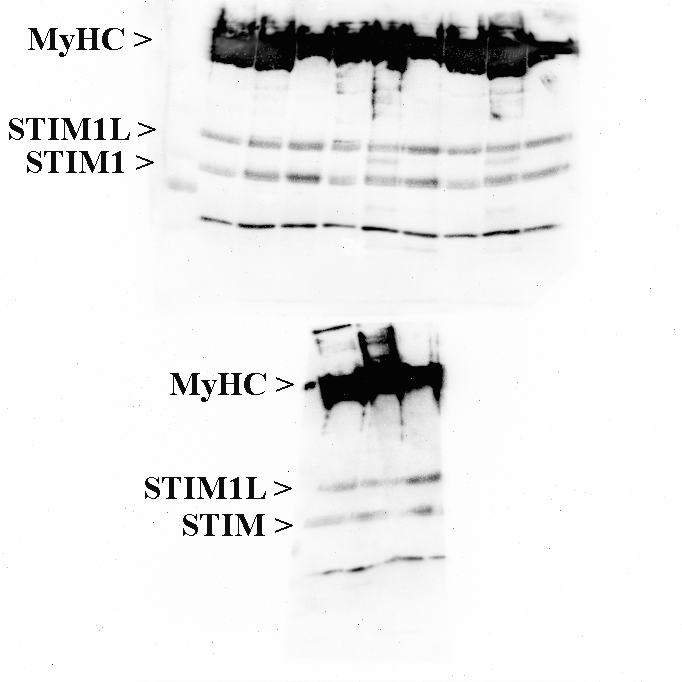

Supplement: Figure 6—source data 2. — Original uncropped western blots showing immunoreactivity of STIM1L, STIM1 and MyHC in EDL, Soleus and EOM muscles from WT mouse N° 1, 2, 3 (TOP) and WT mouse N° 4 (BOTTOM). [file elife-83618-fig6-data2.zip › Figure_6-source_data_2/Figure 6_source data 2.tif]
