# Supplementary material for: Quantitative proteomic analysis of skeletal muscles from wild-type and transgenic mice carrying recessive Ryr1 mutations linked to congenital myopathies
Source: eLife. 2023 Mar 2;12:e83618. doi: 10.7554/eLife.83618 (PMC10038659; doi:10.7554/eLife.83618)
Supplement: Supplementary file 1. — (a) Table showing relative change in protein content between soleus and EDL muscles isolated from WT mice. (b) Table showing relative change in protein content between EOM and EDL muscles isolated from WT mice. (c) Table showing relative change in protein content between EOM and soleus muscles isolated from WT mice. [file elife-83618-supp1.docx]

**Supplementary Material**

**Supplementary 1a:** Relative change in protein content between soleus and EDL muscles isolated from WT mice**.**

|  | **Gene name** | **Protein*** | **Relative content** | **q value** |
| --- | --- | --- | --- | --- |
| **Contractile and sarcomeric proteins** | *Myh 4* | Myosin-4 (MyHC 2b) | 0.01 | 6.14x10^-10^ |
|  | *Actn3* | α-actinin 3 | 0.017 | 6.14x10^-8^ |
|  | *Actn4* | α-actinin 4 | 0.033 | 2.74x10^-8^ |
|  | *Myh3* | Myosin-3 (MyHC emb) | 0.042 | 9.82x10^-5^ |
|  | *Myoz1* | Myozenin 1 | 0.27 | 6.7x10^-7^ |
|  | *Myoz3* | Myozenin 3 | 0.407 | 1.76x10^-6^ |
|  | *Myh6* | Myosin-6 (MyHC-a) | 0.47 | 0.00018 |
|  | *Myom1* | Myomesin-1 | 0.57 | 4.74x10^-6^ |
|  | *Myh14* | Myosin-14 (MyHC non-muscle IIc) | 1.48 | 0.00013 |
|  | *Myh11* | Myosin-11 (MyHC smooth muscle isoform) | 1.57 | 0.0012 |
|  | *Myh10* | Myosin-10 (non-muscle MyHC IIb) | 1.95 | 7.36x10^-6^ |
|  | *Myh13* | MyHC-EO | 2.87 | 0.00068 |
|  | *Des* | Desmin | 4.43 | 2.13x10^-7^ |
|  | *Myot* | Myotilin | 4.43 | 4.71x10^-7^ |
|  | *Myom3* | Myomesin-3 | 7.36 | 4.5x10^-8^ |
|  | *Tnnt1* | Troponin T, slow skeletal muscle (sTnT) | 7.77 | 5.54x10^-5^ |
|  | *TnnI1* | Troponin I, slow skeletal muscle | 32.77 | 6.86x10-^10^ |
|  | *Myoz2* | Myozenin 2 | 53.55 | 6.86x10^-10^ |
|  | *Tnnc1* | Troponin C1, slow skeletal and cardiac muscle) | 84.66 | 2.04x10^-10^ |
|  | *Myh7* | Myosin-7 (MyHC-slow) | 197.0 | 2.49x10^-10^ |
| **ECC** | *Atp2a1* | Sarcoplasmic/endoplasmic reticulum calcium ATPase 1 (SERCA1) | 0.10 | 4.4x10^-7^ |
|  | *Atp2a3* | Sarcoplasmic/endoplasmic reticulum calcium ATPase 3 (SERCA3) | 0.14 | 2.4x10^-6^ |
|  | *Casq1* | Calsequestrin-1 | 0.14 | 5.88x10^-9^ |
|  | *Trdn* | Triadin | 0.20 | 2.99x10^-7^ |
|  | *Stac3* | SH3 and cysteine-rich domain-containing protein 2 (STAC3) | 0.39 | 2.4x10^-6^ |
|  | *Cacna1s* | Voltage dependent L type calcium channel subunit α1s (DHPR α1s) | 0.32** | 1.05x10^-7^ |
|  | *Cacna2d1* | Calcium Voltage-Gated Channel Auxiliary Subunit a2δ 1 | 0.37 | 5.21x10^-7^ |
|  | *Jph1* | Junctophilin-1 | 0.37 | 1.39x10^-5^ |
|  | *Cacnb1* | Voltage dependent L type calcium channel subunit ß1 (DHPR ß1 subunit) | 0.40 | 2.61x10^-7^ |
|  | *Ryr1* | Ryanodine receptor 1 (RyR1) | 0.38** | 3.31x10^-7^ |
|  | *Jph2* | Junctophilin-2 | 0.43 | 1.18x10^-5^ |
|  | *ATP2b4* | Calcium transporting ATPase | 1.38 | 0.0074 |
|  | *Asph* | Aspartyl/asparaginyl ß-hydroxylase (junctin/junctate/aspß-hydroxylase) | 1.44 | 0.0098 |
|  | *Trim72* | Tripartite motif-containing protein 72 (Mitsugumin-53) | 2.68 | 7.98x10^-7^ |
|  | *Casq2* | Calsequestrin-2 | 11.19 | 6.7x10^-10^ |
|  | *Atp2a2* | Sarcoplasmic/endoplasmic reticulum calcium ATPase 2 (SERCA2) | 22.97 | 3.85x10^-9^ |
| **Collagen and ECM** | *Col1a2* | Collagen (I) α2 chain | 0.047 | 0.00081 |
|  | *Col1a1* | Collagen (I) α1 chain | 0.054 | 0.00018 |
|  | *Col18a1* | Collagen (XVIII) α chain | 1.32 | 0.00428 |
|  | *Itgb1* | Integrin β1 | 1.33 | 0.00099 |
|  | *Itga7* | Integrin α7 | 1.39 | 0.00415 |
|  | *Col15a1* | Collagen (XV) α1-chain | 1.42 | 0.00657 |
| **Calcium binding proteins** | *Pvalb* | Parvalbumin | 0.0065 | 2.99x10^-7^ |
|  | *S100a4* | S100 A4 | 0.47 | 0.00046 |
|  | *S100a1* | S100 A1 | 5.18 | 3.11x10^-6^ |
| **Heat shock proteins** | *Dnajb11* | DnaJ homolog subfamily B member 11 (ER-associated HSP40 co-chaperone) | 0.33 | 6.68x10^-5^ |
|  | *Dnajc11* | DnaJ homolog subfamily C member 11 | 0.38 | 0.00073 |
|  | *Dnajc3* | DnaJ homolog subfamily C member 3 | 0.52 | 3.98x10^-5^ |
|  | *Dnajc1* | DnaJ homolog subfamily C member 1 | 1.42 | 0.017 |
|  | *Hspa4* | Hsp family 70 kDa protein 4 | 1.56 | 5.53x10^-5^ |
|  | *Hsp90ab1* | Hsp 90-ß | 1.58 | 2.02x10^-5^ |
|  | *Hspb3* | Hsp ß-3 | 1.58 | 0.00070 |
|  | *Dnaja2* | DnaJ homolog subfamily A member 2 | 1.85 | 0.00025 |
|  | *Hspb2* | Hsp ß-2 | 1.89 | 1.24x10^-5^ |
|  | *Hspa9* | Mitochondrial, stress-70 protein | 1.93 | 6.28x10^-6^ |
|  | *Hspd1* | Mitochondrial, 60 kDa Hsp | 2.08 | 1.14x10^-6^ |
|  | *Hspe1* | Mitochondrial 10 kDa Hsp | 2.48 | 1.01x10^-6^ |
|  | *Hspb7* | Hsp ß-7 | 2.66 | 6.17x10^-7^ |
|  | *Dnaja4* | DnaJ homolog subfamily A member 4 | 3.08 | 6.7x10^-7^ |
|  | *Dnajb4* | DnaJ homolog subfamily B member 4 (Hsp40) | 3.28 | 4.39 x10^-6^ |
|  | *Hspb1* | Hsp ß-1 | 5.43 | 2.17x10^-8^ |
|  | *Hspa1a* | Heat shock 70 kDa protein 1A | 5.75 | 3.05x10^-8^ |
|  | *Hspb6* | Hsp ß- 6 | 16.23 | 2.05x10^-7^ |
| **Proteasomal proteins** | *Psmd6* | 26S proteasome, non-ATPase regulatory subunit 6 | 1.55 | 0.0017 |
|  | *Psmd5* | 26S proteasome, non-ATPase regulatory subunit 5 | 1.59 | 0.0012 |
|  | *Psmd14* | 26S proteasome, non-ATPase regulatory subunit 14 | 1.65 | 0.0199 |
|  | *Psma2* | Proteasome subunit a type-2 | 1.67 | 6.78x10^-5^ |
|  | *Psmd11* | 26S proteasome, non-ATPase regulatory subunit 11 | 1.67 | 3.14x10^-6^ |
|  | *Psmg2* | Proteasome assembly chaperone 2 | 1.68 | 0.0033 |
|  | *Psmb3* | Proteasome subunit ß type-3 | 1.70 | 0.000126 |
| **FK506 binding proteins** | *Fkbp1a* | Peptidyl-prolyl cis-trans isomerase FKBP1A (FKBP12; calstabin-1**)** | 0.57 | 0.00017 |
|  | *Fkbp3* | Peptidyl-prolyl cis-trans isomerase FKBP3 (FK506-binding protein 3) | 1.96 | 0.0047 |
| **Ribosomal proteins** | *Mrps5* | 28S ribosomal Protein S5, mitochondrial | 1.29 | 0.0011 |
|  | *Mrps30* | 28S ribosomal Protein S30, mitochondrial | 1.31 | 0.0046 |
|  | *Rpl22* | 60S Ribosomal Protein L22 | 1.33 | 0.01 |
|  | *Mrpl28* | 39S ribosomal Protein L28, mitochondrial | 1.34 | 0.0021 |
|  | *Rpl18a* | 60S ribosomal Protein L18a | 1.34 | 0.0018 |
|  | *Rps3* | 40S ribosomal Protein S3 | 1.35 | 0.0025 |
|  | *Rpl5* | 60S ribosomal Protein L5 | 1.35 | 0.0091 |
|  | *Mrpl45* | 39S ribosomal Protein L45, mitochondrial | 1.36 | 0.0015 |
|  | *Rpl38* | 60S ribosomal Protein L38 | 1.37 | 0.00079 |
|  | *Rpl12* | 60S ribosomal Protein L12 | 1.37 | 7.46x10^-5^ |
|  | *Rpl24* | 60S ribosomal Protein L24 | 1.38 | 0.0051 |
|  | *Rps11* | 40S ribosomal Protein S11 | 1.38 | 0.0057 |
|  | *Rpsa* | 40S ribosomal Protein SA | 1.39 | 0.0011 |
|  | *Mrpl37* | 39S ribosomal Protein L37, mitochondrial | 1.39 | 0.00018 |
|  | *Mrpl21* | 39S ribosomal Protein L21, mitochondrial | 1.39 | 0.029 |
|  | *Mrps35* | 28S ribosomal Protein S35, mitochondrial | 1.41 | 0.0015 |
|  | *Rps19* | 40S ribosomal Protein S19 | 1.42 | 0.00036 |
|  | *Rpl23a* | 60S ribosomal Protein L23a | 1.43 | 0.00034 |
|  | *Mrpl1* | 39S ribosomal Protein L1, mitochondrial | 1.45 | 0.0012 |
|  | *Rps4x* | 40S ribosomal Protein S4 X-linked | 1.45 | 0.00016 |
|  | *Rps7* | 40S ribosomal Protein S7 | 1.46 | 0.00080 |
|  | *Rpl7* | 60S ribosomal Protein L7 | 1.49 | 0.00015 |
|  | *Rpl31* | 60S ribosomal Protein L31 | 1.50 | 0.016 |
|  | *Mrpl47* | 39S ribosomal Protein L47, mitochondrial | 1.51 | 0.00037 |
|  | *Mrpl48* | 39S ribosomal Protein L48, mitochondrial | 1.51 | 0.00018 |
|  | *Rps2* | 40S ribosomal Protein S2 | 1.57 | 0.00012 |
|  | *Mrpl32* | 39S ribosomal Protein L37, mitochondrial | 1.59 | 9.44x10^-5^ |
|  | *Mrpl19* | 39S ribosomal Protein L19, mitochondrial | 1.59 | 0.0012 |
|  | *Mrps34* | 28S ribosomal Protein S34, mitochondrial | 1.66 | 0.0014 |
|  | *Rpl1* | 60S ribosomal Protein L11 | 1.67 | 1.11x10^-5^ |
|  | *Rps16* | 40S ribosomal Protein S16 | 1.69 | 0.00020 |
|  | *Rpl10* | 60S ribosomal Protein L10 | 1.69 | 3.57x10^-6^ |
|  | *Rpl27* | 60S ribosomal Protein L27 | 1.71 | 0.017 |
|  | *Rpl27a* | 60S ribosomal Protein L27a | 1.77 | 0.0013 |
|  | *Mrpl40* | 39S ribosomal Protein L40 mitochondrial | 1.78 | 8.86x10^-6^ |
|  | *Mrpl38* | 39S ribosomal Protein L38, mitochondrial | 1.83 | 0.00031 |
|  | *Mrpl49* | 39S ribosomal Protein L49, mitochondrial | 1.87 | 7.01x10^-6^ |
|  | *Rps23* | 40S ribosomal Protein S23 | 1.88 | 5.96x10^-5^ |
|  | *Mrpl2* | 39S ribosomal Protein L2, mitochondrial | 1.88 | 3.71x10^-5^ |
|  | *Mrpl53* | 39S ribosomal Protein L53, mitochondrial | 1.90 | 3.25x10^-5^ |
|  | *Rps17* | 40S ribosomal Protein S17 | 1.93 | 0.00021 |
|  | *Mrpl50* | 39S ribosomal Protein L50, mitochondrial | 1.96 | 1.97x10^-6^ |
|  | *Mrpl3* | 39S ribosomal Protein L3, mitochondrial | 1.97 | 3.03x10^-5^ |
|  | *Mrpl13* | 39S ribosomal Protein L13, mitochondrial | 1.97 | 3.03x10^-5^ |
|  | *Rps10* | 40S ribosomal Protein S10 | 2.00 | 0.0057 |
|  | *Mrps7* | 28S ribosomal Protein S7, mitochondrial | 2.07 | 0.00020 |
|  | *Mrps28* | 28S ribosomal Protein S28, mitochondrial | 2.13 | 0.0070 |
|  | *Rps25* | 40S ribosomal Protein S25 | 2.19 | 0.0024 |
|  | *Mrps31* | 28S ribosomal Protein S31, mitochondrial | 2.31 | 7.26x10^-6^ |
|  | *Mrps22* | 28S ribosomal Protein S22, mitochondrial | 2.50 | 1.67x10^-6^ |
|  | *Mrpl16* | 39S ribosomal Protein L16, mitochondrial | 2.66 | 3.21x10^-5^ |
|  | *Mrpl57* | 39S ribosomal Protein L57, mitochondrial | 2.76 | 5.48x10^-7^ |
|  | *Mrps27* | 28S ribosomal Protein S27, mitochondrial | 2.80 | 3.93x10^-6^ |
|  | *Mrps23* | 28S ribosomal Protein S23, mitochondrial | 2.90 | 4.05x10^-5^ |
|  | *Mrpl33* | 39S ribosomal Protein L33, mitochondrial | 4.35 | 3.63x10^-7^ |
|  | *Mrpl43* | 39S ribosomal Protein L34, mitochondrial | 4.63 | 5.26x10^-6^ |
| **Calcium dependent protein kinases** | *Camk2a* | Calcium/calmodulin dependent protein kinase II subunit α | 0.25 | 6.14x10^-8^ |
|  | *Camk2g* | Calcium/calmodulin dependent protein kinase II subunit γ | 0.52 | 0.0011 |
| **Varia** | *Fth1* | Ferritin | 1.64 | 0.00468 |
|  | *Atp1a2* | Na+/K+ ATPase α2 | 1.83 | 3.25x10^-5^ |
|  | *Sod2* | Superoxide dismutase (mitochondrial) | 2.39 | 3.14x10^-6^ |
|  | *Mtor* | Serine-threonine-protein kinase mTOR (Mechanistic target of rapamycin) | 2.49 | 0.0068 |
|  | *Atp1a1* | Na+/K+ ATPase α1 | 3.09 | 2.05x10^-7^ |
|  | *Cat* | Catalase | 3.28 | 2.04x10^-7^ |
|  | *Atp1b1* | Na+/K+ ATPase ß1 | 4.85 | 1.2x10^-5^ |
|  | *Ca3* | Carbonic anhydrase 3 | 12.91 | 0.0060 |
|  | *Mb* | Myoglobin | 21.45 | 8.92x10^-7^ |

*The nomenclature of Proteins is based on that of the UniProtKB database

**These ratio values were calculated based on the absolute concentration determined with the peptides (Table 4).

**Supplementary 1b:** Relative change in protein content between EOM and EDL muscles isolated from WT mice.

|  | **Gene name** | **Protein*** | **Relative content** | **q value** |
| --- | --- | --- | --- | --- |
| **Contractile and sarcomeric proteins** | *Actn3* | α-actinin 3 | 0.021 | 1.6x10^-9^ |
|  | *Myh 4* | Myosin-4 (MyHC 2b) | 0.072 | 5.68x10^-8^ |
|  | *Myh1* | MyHC-2x | 0.61 | 0.0053 |
|  | *Actn4* | α-actinin 4 | 0.13 | 6.10x10^-8^ |
|  | *Myl4* | Myosin light chain 4 | 0.16 | 2.05x10^-8^ |
|  | *Myl1* | Myosin light chain 1 | 0.17 | 1.62x10^-7^ |
|  | *Mylpf* | Myosin light chain phosphorylatable fast muscle | 0.17 | 7.68x10^-7^ |
|  | *Myoz1* | Myozenin 1 | 0.18 | 3.77x10^-9^ |
|  | *Myom1* | Myomesin-1 | 0.31 | 2.16x10^-8^ |
|  | *Myoz3* | Myozenin 3 | 0.49 | 2.60x10^-6^ |
|  | *Myot* | Myotilin | 0.73 | 0.024 |
|  | *Myh14* | Myosin-14 (MyHC non-muscle IIc) | 1.66 | 1.14x10^-5^ |
|  | *Myh10* | Myosin-10 (non-muscle MyHC IIb) | 1.68 | 0.00018 |
|  | *Myh7b* | Myosin heavy chain 7b (cardiac muscle, ß) | 1.91 | 4.90x10^-5^ |
|  | *Des* | Desmin | 1.96 | 1.56x10^-5^ |
|  | *Tnnt2* | Cardiac troponin T | 2.68 | 1.13x10^-5^ |
|  | *Myh11* | Myosin-11 (MyHC smooth muscle isoform) | 2.85 | 0.00 16 |
|  | *Myh2* | Myosin heavy chain -2A | 3.59 | 0.013 |
|  | *Myom3* | Myomesin-3 | 4.72 | 1.0x10^-6^ |
|  | *Myh3* | Myosin heavy chain, embryonic | 8.81 | 0.0014 |
|  | *Myh7* | Myosin-7 (MyHC-slow) | 24.60 | 1.60x10^-7^ |
|  | *Myh13* | MyHC-EO | 29.40 | 1.01x10^-8^ |
|  | *Tnnc1* | Troponin C1, slow skeletal and cardiac muscle) | 31.17 | 9.79x10^-8^ |
| **ECC** | *Stac3* | SH3 and cysteine-rich domain-containing protein 2 (STAC3) | 0.26 | 2.4x10^-6^ |
|  | *Trdn* | Triadin | 0.25 | 2.33x10^-6^ |
|  | *Casq1* | Calsequestrin-1 | 0.28 | 1.17x10^-6^ |
|  | *Jph2* | Junctophilin-2 | 0.31 | 3.57x10^-8^ |
|  | *Jph1* | Junctophilin-1 | 0.34 | 1.02x10^-6^ |
|  | *Cacna1s* | Voltage dependent L type calcium channel subunit a1s (DHPR α1s) | 0.37** | 8.46x10^-8^ |
|  | *Dhrs7c* | Dehydrogenase/reductase SDR family member 7C (SRP-35) | 0.36 | 7.60x10^-6^ |
|  | *Ryr1* | Ryanodine receptor 1 (RyR1) | 0.46** | 2.08x10^-7^ |
|  | *Cacna2d1* | Calcium Voltage-Gated Channel Auxiliary Subunit a2δ 1 | 0.54 | 0.00025 |
|  | *Orai1* | Orai1 | 1.45** | 0.0016 |
|  | *Stim2* | Stromal interaction molecule 2 | 2.30 | 0.032 |
|  | *ATP2b4* | PM Ca^2+^ ATPase protein 4 | 2.53 | 2.56x10^-5^ |
|  | *ATP2b1* | PM Ca^2+^ ATPase | 2.61 | 2.16x10^-8^ |
|  | *Stim1* | Stromal interaction molecule 1 | 2.93** | 1.53x10^-7^ |
|  | *Asph* | Aspartyl/asparaginyl ß-hydroxylase (junctin/junctate/aspß-hydroxylase) | 3.46 | 2.30x10^-8^ |
|  | *Atp2a2* | Sarcoplasmic/endoplasmic reticulum calcium ATPase 2 (SERCA2) | 3.62 | 1.10x10^-8^ |
|  | *Casq2* | Calsequestrin-2 | 21.50 | 1.91x10^-10^ |
| **Collagen and ECM** | *Col24a1* | Collagen (XXIV) α-1 chain | 0.022 | 8.42x10^-7^ |
|  | *Col1a2* | Collagen (I) α2-chain | 0.030 | 2.38x10^-6^ |
|  | *Col1a1* | Collagen (I) α1 chain | 0.030 | 2.57x10^-6^ |
|  | *Col2a1* | Collagen (II) pro-α1 chain | 0.058 | 8.34x10^-7^ |
|  | *Col12a1* | Collagen (XII) α-1 chain | 0.15 | 2.24x10^-5^ |
|  | *Col11a1* | Collagen (XI) α -1 chain | 0.16 | 1.59x10^-6^ |
|  | *Col11a2* | Collagen (XI) pro α -11 chain | 0.19 | 4.15x10^-6^ |
|  | *Col18a1* | Collagen (XVIII) α-1 chain | 1.44 | 0.0041 |
|  | *Col5a2* | Collagen (V) α− 2 chain | 1.60 | 0.00135 |
|  | *Col6a2* | Collagen (VI) α-2 chain | 1.73 | 0.00066 |
|  | *Itgb4* | Integrin ß4 | 1.88 | 0.0021 |
|  | *Itgb6* | Integrin ß6 | 1.93 | 4.60x10^-6^ |
|  | *Col3a1* | Collagen (III) α-1 chain | 1.95 | 0.05 |
|  | *Itgav* | Integrin Subunit α V | 2.00 | 2.64x10^-6^ |
|  | *Col15a1* | Collagen (XV) α-1 chain | 2.14 | 4.42x10^-7^ |
|  | *Itga6* | Integrin Subunit α 6 | 2.35 | 2.78x10^-7^ |
|  | *Col28a1* | Collagen (XXVII) α-1 chain | 2.38 | 0.00078 |
|  | *Col6a1* | Collagen (VI) α-1 chain | 2.59 | 4.22x10^-6^ |
|  | *Col14a1* | Collagen (XIV) α-1 chain | 2.91 | 5.48x10^-7^ |
|  | *Col6a5* | Collagen (VI) α-1 chain | 3.13 | 0.00030 |
|  | *Col6a6* | Collagen (VI) α-6 chain | 3.14 | 1.04x10^-6^ |
|  | *Itga5* | Integrin α 5 | 3.60 | 1.17x10^-7^ |
| **Calcium binding proteins** | *Pvalb* | Parvalbumin | .025 | 2.47x10^-7^ |
|  | *S100a4* | S100 A4 | 0.24 | 0.00078 |
|  | *Calm1* | Calmodulin 1 | 0.53 | 4.66x10^-6^ |
|  | *S100a13* | S100 A13 | 1.50 | 0.00041 |
|  | *S100a11* | S100 A11 | 3.22 | 0.00052 |
|  | *S100a10* | S100 A10 | 3.52 | 1.60x10^-6^ |
|  | *S100a1* | S100 A1 | 17.28 | 1.74x10^-8^ |
| **Heat shock proteins** | *Dnajc11* | DnaJ homolog subfamily C member 11 | 0.43 | 0.0012 |
|  | *Hspb3* | Hsp ß-3 | 0.59 | 0.00064 |
|  | *Dnajb11* | DnaJ homolog subfamily B member 11 (ER-associated HSP40 co-chaperone) | 0.63 | 0.00036 |
|  | *Hspb7* | Hsp ß-7 | 0.64 | 0.0018 |
|  | *Dnajb5* | DnaJ Heat Shock Protein Family (Hsp40) Member B5 | 0.70 | 0.0042 |
|  | *Dnajc1* | DnaJ homolog subfamily C member 1 | 1.55 | 0.0096 |
|  | *Dnajc5* | DnaJ Heat Shock Protein Family (Hsp40) Member C5 | 1.56 | 0.00022 |
|  | *Hspa4* | Hsp family 70 kDa protein 4 | 1.57 | 8.98x10^-6^ |
|  | *Hspa12b* | Heat Shock Protein Family A (Hsp70) Member 12B | 1.70 | 1.02x10^-5^ |
|  | *Hspb1* | Hsp ß-1 | 1.90 | 6.33x10^-6^ |
|  | *Dnaja1* | DnaJ homolog subfamily A member 1 | 1.96 | 9.34x10^-5^ |
|  | *Hspa13* | Heat Shock Protein Family A (Hsp70) Member 13 | 1.97 | 0.00013 |
|  | *Hsp90aa1* | Heat Shock Protein 90 a Family Class A Member 1 | 2.01 | 7.58x10^-6^ |
|  | *Dnaja3* | DNAJ/Hsp40 | 2.01 | 9.49x10^-6^ |
|  | *Dnajc9* | DnaJ Heat Shock Protein Family (Hsp40) Member C9 | 2.02 | 0.00026 |
|  | *Hsph1* | Heat Shock Protein 105 KDa | 2.03 | 1.14x10^-6^ |
|  | *Hspa5* | Heat Shock Protein Family A (Hsp70) Member 5, BiP | 2.05 | 6.72x10^-7^ |
|  | *Hsp90b1* | Heat Shock Protein 90 ß Family Member 1 | 2.28 | 4.01x10^-6^ |
|  | *Hsp90ab1* | Heat Shock Protein 90 a Family Class B Member 1 | 2.42 | 5.93x10^-7^ |
|  | *Hspa1b* | Heat Shock Protein Family A (Hsp70) Member 1B | 2.83 | 1.89x10^-7^ |
|  | *Dnajc28* | DnaJ Heat Shock Protein Family (Hsp40) Member C28 | 3.30 | 3.34x10^-8^ |
|  | *Hspa4l* | Heat Shock Protein Family A (Hsp70) Member 4 Like | 3.15 | 1.83x10^-8^ |
| **Proteasomal proteins** | *Psmb4* | Proteasome subunit ß type-4 | 1.50 | 0.00015 |
|  | *Psmd13* | 26S proteasome, non-ATPase regulatory subunit 13 | 1.52 | 1.60x10^-5^ |
|  | *Psmb3* | Proteasome subunit ß type-3 | 1.63 | 4.83x10^-5^ |
|  | *Psmc4* | Proteasome 20S Subunit ß 3 | 1.63 | 3.0x10^-5^ |
|  | *Psme2* | Proteasome Activator Subunit 2 | 1.65 | 1.13x10^-5^ |
|  | *Psmc5* | Proteasome 26S Subunit, ATPase 5 | 1.68 | 1.63x10^-5^ |
|  | *Psma2* | Proteasome 20S Subunit a 2 | 1.74 | 0.00011 |
|  | *Psme1* | Proteasome Activator Subunit 1 | 1.99 | 1.44x10^-6^ |
|  | *Psmd5* | Proteasome 26S Subunit, Non-ATPase 5, | 2.21 | 7.53x10^-6^ |
|  | *Psme3* | Proteasome Activator Subunit 3 | 2.26 | 1.99x10^-6^ |
| **Ribosomal Proteins** | *Mrps31* | 28S ribosomal Protein S31, mitochondrial | 0.25 | 4.67x10^-7^ |
|  | *Rpl29* | 60S ribosomal Protein L29 | 0.40 | 0.00027 |
|  | *Rps16* | 40S ribosomal Protein S16 | 0.53 | 2.92x10^-6^ |
|  | *Rpl25* | 60S ribosomal Protein L25 | 0.63 | 0.00072 |
|  | *Rps20* | 40S ribosomal Protein L20 | 0.74 | 0.0031 |
|  | *Rpl35a* | 60S ribosomal Protein L35A | 0.70 | 0.049 |
|  | *Mrps33* | 28S ribosomal Protein S33, mitochondrial | 1.26 | 0.040 |
|  | *Mrpl4* | 39S ribosomal Protein L4, mitochondrial | 1.26 | 0.030 |
|  | *Rps13* | 40S ribosomal Protein S13 | 1.30 | 0.0037 |
|  | *Rps9* | 40S ribosomal Protein S9 | 1.34 | 0.0037 |
|  | *Rps23* | 40S ribosomal Protein S23 | 1.35 | 0.00086 |
|  | *Rpl28* | 60S ribosomal Protein L28 | 1.35 | 0.00045 |
|  | *Rpl10a* | 60S ribosomal Protein L10A | 1.36 | 0.00095 |
|  | *Rpl2* | 60S ribosomal Protein L25 | 1.37 | 0.0028 |
|  | *Rpl6* | 60S ribosomal Protein L6 | 1.38 | 0.0013 |
|  | *Rpl34* | 60S ribosomal Protein L34 | 1.38 | 0.0096 |
|  | *Rps10* | 40S ribosomal Protein S10 | 1.39 | 0.0090 |
|  | *Rps19* | 40S ribosomal Protein S19 | 1.40 | 0.0013 |
|  | *Rpl18* | 60S ribosomal Protein L18 | 1.41 | 0.00050 |
|  | *Rps11* | 40S ribosomal Protein S11 | 1.43 | 0.00079 |
|  | *Rps18* | 40S ribosomal Protein S18 | 1.43 | 0.00020 |
|  | *Mrpl50* | 39S ribosomal Protein L50, mitochondrial | 1.44 | 0.0038 |
|  | *Rpl7a* | 60S ribosomal Protein L7A | 1.44 | 0.0038 |
|  | *Rpl23* | 60S ribosomal Protein L23 | 1.46 | 0.0015 |
|  | *Rpl10* | 60S ribosomal Protein L10 | 1.47 | 0.0013 |
|  | *Rpl4* | 60S ribosomal Protein L4 | 1.47 | 0.00066 |
|  | *Mrpl48* | 39S ribosomal Protein L48, mitochondrial | 1.48 | 0.00032 |
|  | *Mrps22* | 28S ribosomal Protein S22, mitochondrial | 1.48 | 0.0022 |
|  | *Rps14* | 40S ribosomal Protein S14 | 1.48 | 0.00087 |
|  | *Rps6kb2* | Ribosomal Protein S6 Kinase B2 | 1.48 | 0.0013 |
|  | *Rps27l* | Ribosomal Protein S27 like | 1.50 | 0.00016 |
|  | *Rpl36a* | 60S ribosomal Protein L36A | 1.50 | 0.0058 |
|  | *Mrpl2* | 39S ribosomal Protein L2, mitochondrial | 1.51 | 1.32x10^-5^ |
|  | *Rps8* | 40S ribosomal Protein S8 | 1.51 | 0.00026 |
|  | *Rps4x* | Ribosomal Protein S4 X-linked | 1.54 | 0.00095 |
|  | *Mrps26* | 28S ribosomal Protein S26, mitochondrial | 1.55 | 0.0010 |
|  | *Rps12* | 40S ribosomal Protein S12 | 1.56 | 0.00011 |
|  | *Mrpl21* | 39S ribosomal Protein L21, mitochondrial | 1.59 | 1.84x10^-5^ |
|  | *Mrpl19* | 39S ribosomal Protein L19, mitochondrial | 1.60 | 0.0022 |
|  | *Rps6ka5* | Ribosomal Protein S6 Kinase A5 | 1.61 | 0.00079 |
|  | *Rpl7* | 60S ribosomal Protein L7 | 1.62 | 2.29x10^-5^ |
|  | *Rpl11* | 60S ribosomal Protein L11 | 1.65 | 0.0050 |
|  | *Mrpl11* | 39S ribosomal Protein L11, mitochondrial | 1.66 | 0.00038 |
|  | *Rpl18a* | 60S ribosomal Protein L18A | 1.68 | 6.94x10^-5^ |
|  | *Mrpl45* | 39S ribosomal Protein L45, mitochondrial | 1.68 | 0.00068 |
|  | *Rpl24* | 60S ribosomal Protein L24 | 1.70 | 0.00010 |
|  | *Mrpl34* | 28S ribosomal Protein S34, mitochondrial | 1.75 | 0.0434 |
|  | *Mrpl3* | 39S ribosomal Protein L3, mitochondrial | 1.77 | 0.00087 |
|  | *Mrpl38* | 39S ribosomal Protein L38, mitochondrial | 1.79 | 0.0013 |
|  | *Rps3* | 40S ribosomal Protein S3 | 1.79 | 2.46x10^-5^ |
|  | *Rplp2* | Ribosomal Protein Lateral Stalk Subunit P2 | 1.80 | 4.17x10^-5^ |
|  | *Mrpl40* | 39S ribosomal Protein L40, mitochondrial | 1.80 | 0.00044 |
|  | *Mrpl55* | 39S ribosomal Protein L55, mitochondrial | 1.80 | 0.0020 |
|  | *Mrpl47* | 39S ribosomal Protein L47, mitochondrial | 1.81 | 5.81x10^-6^ |
|  | *Rps2* | 40S ribosomal Protein S2 | 1.82 | 7.30x10^-6^ |
|  | *Rps26* | 40S ribosomal Protein S26 | 1.87 | 1.81x10^-6^ |
|  | *Mrps5* | 28S ribosomal Protein S5, mitochondrial | 1.88 | 0.00025 |
|  | *Rps6ka4* | Ribosomal Protein S6 Kinase A4 | 1.88 | 7.08x10^-6^ |
|  | *Mrpl13* | 39S ribosomal Protein L13, mitochondrial | 1.90 | 6.22x10^-6^ |
|  | *Mrps18a* | 28S ribosomal Protein S18a, mitochondrial | 1.93 | 1.57x10^-5^ |
|  | *Mrpl58* | 39S ribosomal Protein L58, mitochondrial | 1.98 | 5.03x10^-6^ |
|  | *Mrpl16* | 39S ribosomal Protein L16, mitochondrial | 1.98 | 2.46x10^-5^ |
|  | *Mrps35* | 28S ribosomal Protein S35, mitochondrial | 1.98 | 8.79x10^-7^ |
|  | *Rpsa* | Ribosomal Protein SA | 1.99 | 0.00015 |
|  | *Rpl27a* | 60S ribosomal Protein L27A | 2.01 | 3.72x10^-6^ |
|  | *Rpl38* | 60S ribosomal Protein L38 | 2.02 | 1.39x10^-5^ |
|  | *Mrps7* | 28S ribosomal Protein S7, mitochondrial | 2.02 | 2.94x10^-6^ |
|  | *Rps17* | 40S ribosomal Protein S17 | 2.05 | 3.46x10^-5^ |
|  | *Mrpl53* | 39S ribosomal Protein L53, mitochondrial | 2.05 | 1.83x10^-5^ |
|  | *Mrpl49* | 39S ribosomal Protein L49, mitochondrial | 2.06 | 7.20x10^-5^ |
|  | *Mrps9* | 28S ribosomal Protein S9, mitochondrial | 2.07 | 1.79x10^-6^ |
|  | *Mrps6* | 28S ribosomal Protein S6, mitochondrial | 2.13 | 0.00084 |
|  | *Mrpl44* | 39S ribosomal Protein L44, mitochondrial | 2.13 | 1.75x10^-7^ |
|  | *Mrpl22* | 39S ribosomal Protein L22, mitochondrial | 2.13 | 0.00074 |
|  | *Rpl22* | 60S ribosomal Protein L22 | 2.15 | 0.00010 |
|  | *Rpl3* | 60S ribosomal Protein L3 | 2.18 | 5.69x10^-6^ |
|  | *Mrpl4* | 39S ribosomal Protein L4, mitochondrial | 2.21 | 4.39x10^-5^ |
|  | *Mrpl37* | 39S ribosomal Protein L37, mitochondrial | 2.22 | 6.61x10^-7^ |
|  | *Mrpl17* | 39S ribosomal Protein L17, mitochondrial | 2.24 | 4.06x10^-5^ |
|  | *Rps25* | 40S ribosomal Protein S25 | 2.29 | 2.03x10^-6^ |
|  | *Rpl9* | 60S ribosomal Protein L9 | 2.30 | 3.74x10^-7^ |
|  | *Mrpl9* | 39S ribosomal Protein L9, mitochondrial | 2.30 | 1.80x10^-6^ |
|  | *Mrpl39* | 39S ribosomal Protein L39, mitochondrial | 2.32 | 6.59x10^-7^ |
|  | *Rplp0* | Ribosomal Protein Lateral Stalk Subunit P0 | 2.40 | 6.31x10^-6^ |
|  | *Rpl5* | 60S ribosomal Protein L5 | 2.41 | 3.12x10^-7^ |
|  | *Mrpl12* | 39S ribosomal Protein L12, mitochondrial | 2.43 | 4.56x10^-7^ |
|  | *Mrps25* | 28S ribosomal Protein S25, mitochondrial | 2.42 | 2.83x10^-7^ |
|  | *Mrpl15* | 39S ribosomal Protein L15, mitochondrial | 2.43 | 1.07x10^-6^ |
|  | *Mrps30* | 28S ribosomal Protein S30, mitochondrial | 2.58 | 3.21x10^-5^ |
|  | *Mrps18b* | 28S ribosomal Protein S18b, mitochondrial | 2.66 | 6.66x10^-6^ |
|  | *Mrps27* | 28S ribosomal Protein S27 mitochondrial | 2.82 | 3.91x10^-8^ |
|  | *Mrpl24* | 39S ribosomal Protein L24, mitochondrial | 2.86 | 4.48x10^-6^ |
|  | *Mrps23* | 28S ribosomal Protein S23, mitochondrial | 2.94 | 1.78x10^-6^ |
|  | *Mrpl57* | 39S ribosomal Protein L57, mitochondrial | 2.97 | 3.78x10^-7^ |
|  | *Mrpl1* | 39S ribosomal Protein L1, mitochondrial | 2.98 | 4.40x10^-8^ |
|  | *Mrps16* | 28S ribosomal Protein S16, mitochondrial | 2.98 | 0.00030 |
|  | *Mrpl10* | 39S ribosomal Protein L10, mitochondrial | 3.15 | 2.80x10^-6^ |
|  | *Rps7* | 40S ribosomal Protein S7 | 3.16 | 3.11x10^-6^ |
|  | *Mrpl28* | 39S ribosomal Protein L28, mitochondrial | 3.18 | 2.06x10^-6^ |
|  | *Mrpl41* | 39S ribosomal Protein L41, mitochondrial | 3.44 | 3.91x10^-8^ |
|  | *Mrpl46* | 39S ribosomal Protein L46, mitochondrial | 3.94 | 4.29x10^-8^ |
|  | *Mrps36* | 28S ribosomal Protein S36, mitochondrial | 4.22 | 2.93x10^-6^ |
|  | *Mrps17* | 28S ribosomal Protein S17, mitochondrial | 5.54 | 1.01x10^-8^ |
| **FK506 binding proteins** | *Fkbp1a* | Peptidyl-prolyl cis-trans isomerase FKBP1A (FKBP12; calstabin-1**)** | 0.52 | 3.92x10^-5^ |
|  | *Fkbp15* | Peptidyl-prolyl cis-trans isomerase FKBP3 (FK506-binding protein 15) | 2.31 | 2.44x10^-5^ |
| **Calcium dependent protein kinases** | *Camk2a* | Calcium/calmodulin dependent protein kinase II subunit α | 0.31 | 3.47x10^-8^ |
|  | *Camk2g* | Calcium/calmodulin dependent protein kinase II subunit γ | 0.62 | 0.00038 |
|  | *Camk2d* | Calcium/calmodulin dependent protein kinase II subunit δ | 1.57 | 0.00029 |
| **Varia** | *Ca3* | Carbonic anhydrase 3 | 0.089 | 0.0027 |
|  | *Atp1b2* | Na+/K+ ATPase ß2 | 0.20 | 1.69x10^-7^ |
|  | *Mtor* | Serine-threonine-protein kinase mTOR (Mechanistic target of rapamycin) | 1.76 | 3.44x10^-5^ |
|  | *Cat* | Catalase | 1.98 | 0.00027 |
|  | *Mb* | Myoglobin | 3.85 | 7.40x10^-5^ |
|  | *Atp1a2* | Na+/K+ ATPase α 2 | 4.41 | 1.60x10^-9^ |
|  | *Sod2* | Superoxide dismutase (mitochondrial) | 6.60 | 1.28x10^-8^ |
|  | *Atp1a3* | Na+/K+ ATPase α 3 | 6.79 | 7.67x10^-9^ |
|  | *Atp1a1* | Na+/K+ ATPase α 1 | 7.69 | 1.60x10^-9^ |

*The nomenclature of Proteins is based on that of the UniProtKB database

**These ratio values were calculated based on the absolute concentration determined with the peptides (Table 4).

**Supplementary 1c:** Relative change in protein content between EOM and soleus muscles isolated from WT mice.

|  | **Gene name** | **Protein*** | **Relative content** | **q value** |
| --- | --- | --- | --- | --- |
| **Contractile and sarcomeric proteins** | *Myh 7* | Myosin-7 (MyHC-slow) | 0.0096 | 2.62x10^-9^ |
|  | *Myl2* | Myosin light chain 2 | 0.010 | 1.39x10^-8^ |
|  | *Actn2* | α -actinin 2 | 0.017 | 5.26x10^-8^ |
|  | *Myoz2* | Myozenin-2 | 0.017 | 8.31x10^-10^ |
|  | *Actn1* | α -actinin 1 | 0.012 | 0.0010 |
|  | *Myh6* | Myosin-6 (MyHC-a) | 0.012 | 2.14x10^-11^ |
|  | *Myl10* | Myosin light chain 10 | 0.019 | 6.30x10^-9^ |
|  | *Myh2* | Myosin heavy chain -2A | 0.02 | 1.48x10^-8^ |
|  | *Tnnc1* | Troponin C1, slow skeletal and cardiac muscle) | 0.025 | 5.79x10^-5^ |
|  | *Myl3* | Myosin light chain 3 | 0.029 | 4.66x10^-9^ |
|  | *Tnni1* | Troponin I1, Slow Skeletal Type | 0.049 | 1.38x10^-8^ |
|  | *Tnnt1* | Troponin T, slow skeletal muscle (sTnT) | 0.049 | 3.02x10^-6^ |
|  | *Myot* | Myotilin | 0.14 | 9.80x10^-8^ |
|  | *Myh8* | MyHC-fetal and embryonic | 0.15 | 1.63x10^-6^ |
|  | *Myom3* | Myomesin-3 | 0.29 | 3.77x10^-8^ |
|  | *Des* | Desmin | 0.40 | 9.36x10^-6^ |
|  | *Tnnt3* | Troponin T3, Fast Skeletal | 0.53 | 0.0036 |
|  | *Myom1* | Myomesin-1 | 0.54 | 7.23x10^-5^ |
|  | *Myh4* | Myosin-4 (MyHC 2b) | 0.67 | 0.0062 |
|  | *Myh11* | Myosin-11 (MyHC smooth muscle isoform) | 1.47 | 0.014 |
|  | *Myoz3* | Myozenin 3 | 1.53 | 0.0022 |
|  | *Myl9* | Myosin light chain 9 | 1.66 | 0.0018 |
|  | *Myl4* | Myosin light chain 4 | 1.86 | 0.012 |
|  | *Mylk* | myosin light chain kinase | 1.88 | 0.00016 |
|  | *Myl6b* | myosin alkali light chain, fast skeletal muscle | 1.90 | 0.0082 |
|  | *Myh7b* | Myosin-7 (MyHC-slow) | 2.06 | 8.48x10^-6^ |
|  | *Actn3* | α -actinin 3 | 2.83 | 0.0042 |
|  | *Myl1* | Myosin light chain 1 | 2.91 | 0.00036 |
|  | *Tnnt2* | Cardiac troponin T | 3.10 | 3.56x10^-5^ |
|  | *Myh13* | MyHC-EO | 19.57 | 2.62x10^-9^ |
|  | *Myh3* | Myosin heavy chain, embryonic | 49.06 | 2.86x10^-8^ |
| **ECC** | *Atp2a2* | Sarcoplasmic/endoplasmic reticulum calcium ATPase 2 (SERCA2) | 0.041 | 1.38x10^-8^ |
|  | *Trim72* | Tripartite motif-containing protein 72 (Mitsugumin-53) | 0.40 | 1.16x10^-5^ |
|  | *Jph2* | Junctophilin-2 | 0.62 | 0.00084 |
|  | *Cacnb1* | Calcium Voltage-Gated Channel Auxiliary Subunit ß 1 | 1.43 | 0.00059 |
|  | *Jph1* | Junctophilin-1 | 1.44 | 0.0045 |
|  | *Cacna2d1* | Calcium Voltage-Gated Channel Auxiliary Subunit a2δ 1 | 1.51 | 0.0024 |
|  | *ATP2b4* | Calcium transporting ATPase | 1.58 | 0.00050 |
|  | *Trdn* | Triadin | 1.74 | 0.0050 |
|  | *Atp2b1* | PM Ca^2+^ ATPase | 2.29 | 4.41x10^-6^ |
|  | *Casq1* | Calsequestrin-1 | 2.34 | 0.00011 |
|  | *Stim1* | Stromal Interaction Molecule 1 | 2.45** | 6.84x10^-7^ |
|  | *Asph* | Aspartyl/asparaginyl ß-hydroxylase (junctin/junctate/aspß-hydroxylase) | 3.01 | 2.59x10^-6^ |
|  | *Atp2a3* | Sarcoplasmic/endoplasmic reticulum calcium ATPase 3 (SERCA3) | 7.02 | 7.63x10^-7^ |
|  | *Atp1a3* | Na+/K+ ATPase α3 subunit | 7.26 | 1.39x10^-8^ |
|  | *Atp2a1* | Sarcoplasmic/endoplasmic reticulum calcium ATPase 1 (SERCA1) | 8.37 | 2.73x10^-7^ |
| **Collagen and ECM** | *Col11a2* | Collagen (XI) pro α-11 chain | 0.086 | 6.97x10^-5^ |
|  | *Col12a1* | Collagen (XII) α-1 chain | 0.32 | 0.00018 |
|  | *Col2a1* | Collagen (II) pro-α 1 chain | 0.62 | 0.00018 |
|  | *Itga5* | Integrin α 5 | 1.59 | 0.0031 |
|  | *Itgb2* | Integrin ß2 | 1.79 | 0.0087 |
|  | *Itga6* | Integrin α 6 | 2.07 | 1.94x10^-5^ |
|  | *Col15a1* | Collagen (XV) α1 chain | 2.00 | 0.0035 |
|  | *Col6a2* | Collagen (VI) α2 chain | 2.12 | 0.00012 |
|  | *Col6a1* | Collagen (VI) α1 chain | 2.20 | 0.00068 |
|  | *Col3a1* | Collagen (III) α1 chain | 2.33 | 0.00064 |
|  | *Col4a1* | Collagen (IV) α1 chain | 2.52 | 3.14x10^-5^ |
|  | *Col5a1* | Collagen (V) α1 chain | 2.60 | 0.0033 |
|  | *Col28a1* | Collagen (XXVIII) α1 chain | 3.28 | 9.60x10^-6^ |
|  | *Col6a5* | Collagen (VI) α 5 chain | 4.00 | 1.91x10^-7^ |
|  | *Itgb4* | Integrin β 4 | 3.32 | 2.35x10^-5^ |
|  | *Col6a6* | Collagen (VI) α 6 chain | 6.87 | 3.16x10^-10^ |
| **Ribosomal proteins** | *Rpl27* | 60S ribosomal Protein L27 | 0.51 | 0.045 |
|  | *Mrpl33* | 39S ribosomal Protein L33, mitochondrial | 0.52 | 0.00094 |
|  | *Mrpl38* | 39S ribosomal Protein L38, mitochondrial | 0.60 | 0.0047 |
|  | *Rps25* | 40S ribosomal Protein S25 | 0.61 | 2.03x10^-6^ |
|  | *Mrpl50* | 39S ribosomal Protein L50, mitochondrial | 0.67 | 0.0081 |
|  | *Rps6ka3* | Ribosomal Protein 6 Kinase A3 | 0.67 | 0.036 |
|  | *Rps6kb2* | Ribosomal Protein 6 Kinase B2 | 0.70 | 0.0045 |
|  | *Mrpl43* | 39S ribosomal Protein L43, mitochondrial | 0.72 | 0.0041 |
|  | *Rpl10* | 60S ribosomal Protein L10 | 0.74 | 0.011 |
|  | *Rps3* | 40S ribosomal Protein S3 | 1.27 | 0.028 |
|  | *Mrpl21* | 39S ribosomal Protein L21, mitochondrial | 1.27 | 0.030 |
|  | *Mrps27* | 40S ribosomal Protein S27, mitochondrial | 1.27 | 0.015 |
|  | *Mrpl44* | 39S ribosomal Protein L44, mitochondrial | 1.28 | 0.0060 |
|  | *Mrps22* | 40S ribosomal Protein S22, mitochondrial | 1.29 | 0.013 |
|  | *Rps4x* | Ribosomal protein S4 X-linked | 1.30 | 0.020 |
|  | *Mrps9* | 4S0 ribosomal Protein S9, mitochondrial | 1.32 | 0.043 |
|  | *Rpl13a* | 60S ribosomal Protein L13A | 1.32 | 0.047 |
|  | *Rpl23a* | 60S ribosomal Protein L23A | 1.35 | 0.0094 |
|  | *Rps8* | 40S ribosomal Protein S8 | 1.36 | 0.018 |
|  | *Mrps5* | 4S0 ribosomal Protein S5, mitochondrial | 1.36 | 0.045 |
|  | *Mrpl37a* | 39S ribosomal Protein L37A, mitochondrial | 1.37 | 0.019 |
|  | *Rpl38* | 60S ribosomal Protein L38 | 1.39 | 0.023 |
|  | *Mrps30* | 40S ribosomal Protein S30, mitochondrial | 1.41 | 0.0050 |
|  | *Rps21* | 40S ribosomal Protein S21 | 1.46 | 0.024 |
|  | *Mrps23* | 4S0 ribosomal Protein S23, mitochondrial | 1.47 | 0.0046 |
|  | *Mrps7* | 4S0 ribosomal Protein S7, mitochondrial | 1.48 | 0.0025 |
|  | *Mrps35* | 4S0 ribosomal Protein S35, mitochondrial | 1.55 | 0.00095 |
|  | *Mrpl2* | 39S ribosomal Protein L2, mitochondrial | 1.64 | 0.00065 |
|  | *Mrps16* | 4S0 ribosomal Protein S16, mitochondrial | 1.76 | 0.0098 |
|  | *Mrps36* | 4S0 ribosomal Protein S36, mitochondrial | 1.86 | 1.21x10^-5^ |
|  | *Mrpl27* | 39S ribosomal Protein L27, mitochondrial | 1.93 | 0.00040 |
|  | *Rps6ka5* | Ribosomal Protein S6 Kinase A5 | 2.36 | 5.23x10^-5^ |
|  | *Mrpl42* | 39S ribosomal Protein L42, mitochondrial | 3.07 | 1.81x10^-5^ |
| **Calcium binding proteins** | *S100a11* | S100 A11 | 1.44 | 0.015 |
|  | *Calm1* | Calmodulin 1 | 1.47 | 0.0012 |
|  | *S100a13* | S100 A13 | 2.53 | 0.00069 |
|  | *S100a1* | S100 A1 | 2.61 | 0.0018 |
|  | *S100a6* | S100 A6 | 2.62 | 0.0027 |
|  | *S100a10* | S100 A10 | 3.04 | 5.72x10^-6^ |
|  | *Pvalb* | Parvalbumin | 14.06 | 1.52x10^-5^ |
| **Heat shock proteins** | *Hspb7* | Heat Shock Protein Family B (small) member 7 | 0.13 | 3.46x10^-9^ |
|  | *Hspb6* | Hsp ß- 6 | 0.20 | 6.36x10^-5^ |
|  | *Dnajb4* | DnaJ Heat Shock Protein Family (Hsp40) Member B4, | 0.20 | 2.44x10^-7^ |
|  | *Hspb1* | Hsp ß-1 | 0.29 | 1.67x10^-6^ |
|  | *Dnaja4* | DnaJ homolog subfamily A member 4 | 0.34 | 5.07x10^-6^ |
|  | *Hspb3* | Hsp ß-3 | 0.37 | 2.54x10^-5^ |
|  | *Hspb2* | Hsp ß-2 | 0.40 | 8.13x10^-7^ |
|  | *Hspa1b* | Heat Shock Protein Family A (Hsp70) Member 1B | 0.46 | 9.46x10^-6^ |
|  | *Hspa1l* | Heat Shock Protein Family A (Hsp70) Member 1 Like | 0.55 | 0.0066 |
|  | *Dnaja2* | DnaJ homolog subfamily A member 2 | 0.59 | 5.76x10^-5^ |
|  | *Hspa8* | Heat shock 70 kDa protein 8 | 0.59 | 0.0052 |
|  | *Dnajb5* | DnaJ Heat Shock Protein Family (Hsp40) Member B5 | 0.62 | 0.00039 |
|  | *Dnajc9* | DnaJ homolog subfamily C member 9 | 1.31 | 0.042 |
|  | *Dnaja1* | DnaJ homolog subfamily A member 1 | 1.34 | 0.039 |
|  | *Hspa12b* | Heat Shock Protein Family A (Hsp70) Member 12B | 1.45 | 0.0022 |
|  | *Dnajb1* | DnaJ Heat Shock Protein Family (Hsp40) Member B1 | 1.50 | 0.046 |
|  | *Dnajc8* | DnaJ Heat Shock Protein Family (Hsp40) Member C8 | 1.54 | 0.0017 |
|  | *Hspa8* | Heat shock 70 kDa protein 8 | 1.60 | 0.0029 |
|  | *Hspa5* | Heat Shock Protein Family A (Hsp70) Member 5 (BiP) | 1.64 | 0.00018 |
|  | *Dnajc3* | DnaJ Heat Shock Protein Family (Hsp40) Member C3 | 1.66 | 3.44x10^-5^ |
|  | *Dnajc28* | DnaJ Heat Shock Protein Family (Hsp40) Member C28 | 1.66 | 7.46x10^-5^ |
|  | *Dnajc25* | DnaJ Heat Shock Protein Family (Hsp40) Member C25 | 1.71 | 0.0043 |
|  | *Hsph1* | Heat shock protein 105 kDa | 1.79 | 9.71x10^-5^ |
|  | *Dnajb12* | DnaJ Heat Shock Protein Family (Hsp40) Member B12 | 1.86 | 0.00017 |
|  | *Hsp90aa1* | Heat Shock Protein 90 a Family Class A Member 1 | 1.93 | 2.34x10^-5^ |
|  | *Hspa13* | Heat Shock Protein Family A (Hsp70) Member 13 | 1.97 | 1.78x10^-5^ |
|  | *Hspa90b1* | Heat Shock Protein 90 ß Family Member 1 | 2.00 | 1.59x10^-5^ |
|  | *Hspe1* | Heat Shock Protein Family E (Hsp10) Member 1) | 2.00 | 0.00022 |
|  | *Hspd1* | Mitochondrial, 60 kDa Hsp | 2.14 | 4.71x10^-6^ |
|  | *Dnajb11* | DnaJ homolog subfamily B member 11 (ER-associated HSP40 co-chaperone) | 2.20 | 4.95x10^-6^ |
|  | *Hspa4l* | Heat Shock Protein Family A (Hsp70) Member 4 Like | 3.27 | 4.16x10^-6^ |
|  | *Hspa12a* | Heat Shock Protein Family A (Hsp70) Member 12A | 7.67 | 6.15x10^-8^ |
| **Proteasomal proteins** | *Psmd4* | Proteasome 26S Subunit Ubiquitin Receptor, Non-ATPase 4 | 0.55 | 0.0019 |
|  | *Psmd14* | 26S proteasome, non-ATPase regulatory subunit 14 | 0.58 | 0.00063 |
|  | *Psmd12* | Proteasome 26S Subunit, Non-ATPase 12 | 0.60 | 0.00065 |
|  | *Psmd7* | Proteasome 26S Subunit, Non-ATPase 7 | 0.61 | 0.00030 |
|  | *Psmg2* | Proteasome assembly chaperone 2 | 0.70 | 0.0089 |
|  | *Psmd11* | 26S proteasome, non-ATPase regulatory subunit 11 | 0.71 | 0.00071 |
|  | *Psma5* | Proteasome 20S Subunit a 5 | 0.72 | 0.0083 |
|  | *Psmb7* | Proteasome 20S Subunit ß 7 | 0.74 | 0.010 |
|  | *Psme3* | Proteasome Activator Subunit 3 | 1.50 | 0.029 |
| **FK506 binding proteins** | *Fkbp7* | Peptidyl-prolyl cis-trans isomerase FKBP7 (FK506-binding protein 7) | 1.31 | 0.046 |
|  | *Fkbp4* | Peptidyl-prolyl cis-trans isomerase FKBP4 (FK506-binding protein 4) | 1.34 | 0.0095 |
|  | *Fkbp8* | Peptidyl-prolyl cis-trans isomerase FKBP8 (FK506-binding protein 8) | 1.41 | 0.041 |
|  | *Fkbp9* | Peptidyl-prolyl cis-trans isomerase FKBP9 (FK506-binding protein 9) | 1.43 | 0.0062 |
|  | *Fkbp5* | Peptidyl-prolyl cis-trans isomerase FKBP5 (FK506-binding protein 5) | 1.57 | 0.046 |
|  | *Fkbp2* | Peptidyl-prolyl cis-trans isomerase FKBP2 (FK506-binding protein 2) | 2.05 | 0.0029 |
|  | *Fkbp3* | Peptidyl-prolyl cis-trans isomerase FKBP3 (FK506-binding protein 3) | 0.50 | 3.09x10^-5^ |
| **Calcium dependent protein kinases** | *Camk2b* | Calcium/calmodulin dependent protein kinase II subunit ß | 1.37 | 0.036 |
|  | *Camk2a* | Calcium/calmodulin dependent protein kinase II subunit α | 1.52 | 0.00050 |
|  | *Camk2d* | Calcium/calmodulin dependent protein kinase II subunit δ | 1.58 | 8.68x10^-5^ |
|  | *Camk2g* | Calcium/calmodulin dependent protein kinase II subunit γ | 2.08 | 0.031 |
| **Varia** | *Ca3* | Carbonic anhydrase 3 | 0.0041 | 6.84x10^-12^ |
|  | *Mb* | Myoglobin | 0.14 | 1.78x10^-6^ |
|  | *Atb1b4* | Na+/K+ ATPase ß4 | 0.55 | 0.0021 |
|  | *Cat* | Catalase | 0.57 | 0.0070 |
|  | *Sod2* | Superoxide dismutase (mitochondrial) | 1.82 | 0.012 |
|  | *Fth1* | Ferritin | 1.88 | 0.0098 |
|  | *Atp1b1* | Na+/K+ ATPase ß1 | 2.18 | 0.00017 |
|  | *Atp1b3* | Na+/K+ ATPase ß3 | 2.34 | 4.95x10^-6^ |
|  | *Atp1a1* | Na+/K+ ATPase α1 | 2.39 | 4.12x10^-7^ |
|  | *Sod3* | Superoxide dismutase Zn-Cu | 2.46 | 0.00049 |
|  | *Atp1a2* | Na+/K+ ATPase α2 | 2.73 | 3.09x10^-6^ |
|  | *Atp1a3* | Na+/K+ ATPase α3 | 7.26 | 1.39x10^-8^ |

*The nomenclature of Proteins is based on that of the UniProtKB database

**These ratio values were calculated based on the absolute concentration determined with the peptides (Table 4).
